# Cross-cultural adaptation, reliability and validity of the persian version of the functional assessment scale for acute hamstring injuries

**DOI:** 10.1186/s13018-024-05252-3

**Published:** 2024-11-26

**Authors:** Mohammad Hadadi, Farzaneh Haghighat

**Affiliations:** 1 Department of Orthotics and Prosthetics, School of Rehabilitation Sciences, Shiraz University of Medical Sciences, Shiraz, Iran; 2 Orthopedic & Rehabilitation Research Center, Shiraz University of Medical Sciences, Chamran Blvd, Shiraz, Iran

**Keywords:** Questionnaire, Hamstring, Acute injury, Validity, Psychometric properties, Reliability

## Abstract

**Background:**

The Functional Assessment Scale for Acute Hamstring Injuries (FASH) which measure symptom’s severity and impact on physical activity and sports ability in individuals with acute hamstring muscle injury, is not available in Persian. The aim of this study was to translate and cross-culturally adapt the FASH questionnaire to Persian and to assess the psychometric properties of the translated version.

**Methods:**

The Persian-translation and cross-cultural adaptation processes were based on World Health Organization method. A total of 160 participants compromising of four groups: (1) acute hamstring injury (*N* = 40), (2) other lower extremity injury (*N* = 40), (3) risk of acute injury (*N* = 40), and (4) healthy control (*N* = 40) were recruited to complete the Persian version of FASH (FASH-P) questionnaire twice with an interval of 48–60 h. The Short Form Health Survey (SF-36) and Lower Extremity Functional Scale (LEFS) were used to assess the criterion validity. Test-retest reliability, internal consistency, criterion validity, Dimensionality and floor/ceiling effects were evaluated to assess the FASH-P psychometric properties.

**Results:**

The FASH questionnaire were translated to Persian without any major problems. The FASH-P showed excellent power of differentiation because the scores were significantly different among the four groups. Regarding psychometric performances, excellent test–retest reliability (Intraclass correlation coefficient of 0.997) and a high level of internal consistency (Cronbach’s alpha of 0.966) were observed. The FASH-P total score was significantly correlated with SF-36 and LEFS questionnaires representing an excellent criterion validity. No floor or ceiling effect were found for total score in Hamstring muscle injury group.

**Conclusions:**

Due to the excellent psychometric performance, the FASH-P can be used as a reliable and valid tool for evaluating the severity of symptoms and sports ability in Persian-speaking patients with hamstring muscle injury.

## Background

Hamstring muscle injuries (HMIs) are the most common muscle injury in sports involving high-speed running, jumping, acceleration, deceleration, and rapid change of direction such as soccer (football), Australian football, rugby, American football and track and field [1–8]. The estimated rate of incidence was shown to be 3–4.1 per 1000 h of competition and 0.4–0.5 per 1000 h of training; An average increase of 4% annually has been reported [9, 10]. Eccentric contraction at high velocity and slow stretching at outer range of motion are the main mechanisms of HMI in above-mentioned sports [6].

Hamstring muscle injuries often cause prolonged symptoms, poor healing responses and high rate of recurrences which in turn result in significant time-loss from competition and training [1, 6, 9, 11–15] and high costs related to the treatment and lost player payments [16]. The recurrent hamstring injury is usually more severe than the initial injury and results in a longer absence from play [2, 17]. The most prominent risk factor for hamstring muscle re-injury is a previous injury of this muscle probably due to incomplete recovery and/or inadequate rehabilitation [18, 19]. Despite these findings, HMI and its recurrence-related factors are poorly understood and there is no consensus relating the right time to return to sport after the injury [20]. There is therefore an increasing need to develop a valid instrument to assess the severity of the lesion and its evolution until return to sport, evaluate the treatment efficacy, and facilitate comparative research. The only HMI specific questionnaire, the Functional Assessment Scale for Acute Hamstring Injuries (FASH), has been developed and validated by Malliaropoulos et al. in 2014 [21]. This instrument grades the HMI severity and its impact on physical activity and sports ability. This self-reported and disease-specific questionnaire is available in Greek and English [21], German [22], French [23] and Spanish [24] languages. To the best of the authors’ knowledge, however, the FASH is not available in Persian. Therefore, the objctive of this study was to translate and cross-culturally adapt the FASH questionnaire into Persian and the measurement of the psychometric performance of the translated version.

## Methods

Translation and cross-cultural adaptation process.

The FASH questionnaire was translated and cross-culturally adapted into Persian according to the World Health Organization (WHO) method [25] using the following steps:

1. Forward translation: The original version of FASH questionnaire (English language) was translated into Persian three times by three independent native Persian-speaking translators while they were asked to provide a list of alternative translations for some words, phrases or sentences in the questionnaire. After the meeting between translators, an agreement on the initial forward translations was obtained and the synthesized version of three translations was provided.

2. Backward translation: a fourth translator, a native English speaker, back-translated the Persian translation into English while blinded to the original version.

3. Comparing the back translation with the original version: A discussion between the translators took place to compare the back translation with the original English version and modify it regarding cultural differences or linguistic errors until a consensus agreement was obtained. The back translation was also sent to the FASH developer to express his opinion.

4. Pre-test: The consensus document was completed by a population of 20 asymptomatic subjects to test their ability to understand the items and determine any difficulty.

### Participants

This study was approved by the Institutional Review Board (IRB) of the authors’ affiliated institution. After signing an informed consent, 160 participants were recruited for the study compromising 4 groups (40 people in each): a control group (healthy young athletes), a group at risk of acute hamstring injury (athletes of sport teams considered at risk for HMI including soccer, Futsal, and running), a group with lower extremity musculoskeletal injuries other than acute hamstring injury (including anterior cruciate ligament injury, groin pain, ankle injury and knee meniscal tears), and a group with acute hamstring injury (athletes with a definite diagnosis of acute hamstring injury recruited from sport physiotherapy centers, hospitals and sports clubs). Overall inclusion criteria were: age over 18 years and reading and writing literacy in Persian. Overall exclusion criteria were: pregnancy, spinal symptoms and not speaking Persian. The recruitment criteria are detailed in Table 1 [23].

**Table 1:** The recruitment criteria

Group	Description	Inclusion criteria	Exclusion criteria
Acute hamstring muscle injury	Athletes with a confirmed diagnosis of an acute hamstring muscle injury	Acute pain, local sensitivity to palpation at the injured area, pain provocation on resisted knee flexion, resisted hip extension and passive hip flexion with the knee extend, Objective diagnosis of hamstring muscle injury by medical imaging within 24 to 36 h after injury, hamstring muscle injury occurred during training or matches	Uncertain diagnosis of the lesion, bilateral lesion, an old lesion suspected or verified by palpating the posterior thigh muscles, Muscle damage induced by direct external trauma, pain on palpation of the hamstring muscles insertions, avulsion of one or more heads of hamstring muscle, low back pain or sciatica
At risk	Athletes practicing sports that are well-known to expose them to a higher risk of hamstring muscle injury (e.g., soccer, Futsal, and running)	No specific criteria	Any type of leg pain, functional deficit during physical activity, Other lower limb injurie
Other pathology group	Athletes presenting a lesion of the lower limbs (including anterior cruciate ligament injury, groin pain, ankle injury and knee meniscal tears)	No specific criteria	No specific criteria
Control group	Active healthy subjects without any particular risk	No specific criteria	Any type of leg pain, functional deficit during physical activity, Other lower limb injurie

### Assessment of psychometric properties

Once the FASH translation was carried out, the translated questionnaire was submitted to all subjects between April and October 2023. All participants completed the Persian version of FASH questionnaire (FASH-P) twice with an interval of 48–60 h. This time interval of test-retest was chosen short enough to ensure that no clinical change had occurred [4]. Participants were generally advised to keep their daily routines consistent and avoid making significant lifestyle changes, such as initiating therapeutic interventions to ensure that the responses reflect true changes rather than variations in daily activities.

Two other questionnaires, Short‑Form health survey (SF‑36) and Lower Extremity Functional Scale (LEFS) were also submitted to the participants.

### Internal consistency

In order to estimate the internal consistency, i.e. the homogeneity across items of the FASH-P, the Cronbach’s coefficient alpha was calculated. The obtained scores from first administration of FASH-P were used to calculate this value. The alpha value varies between 0 and 1, and an adequate level of internal consistency is obtained when alpha value ranges from 0.70 to 0.95 [26]. Additionally, in the HMI group to investigate the impact of each item on Cronbach’s alpha, the scores were recalculated with each item dropped from the scale.

### Test-retest reliability

For the purpose of assessing the stability of the Persian version of FASH-P over time, test-retest reliability was evaluated by computing the intra-class correlation coefficient (ICC). In this regard, the results of completing the FASH-P twice, separated by 48–60 h were used. The Munro’s classification was considered to interpret ICC values: The correlation coefficient of 0.26 to 0.49 (low), 0.50 to 0.69 (moderate), 0.70 to 0.89 (high), and 0.90 to 1.00 (very high) [27]. The standard error of measurement (SEM) and the minimal detectable change (MDC) were also calculated to assess the measurement error. The SEM indicates the precision of a measurement, while the MDC indicates the minimum change attributable to measurement errors.

### Criterion validity

In order to assess the criterion validity, the association between the scores obtained from the FASH-P and those obtained from SF-36 [28] and lower limb functional index (LLFI) [29] were calculated. The correlation coefficient of less than 0.30, 0.30–0.6 and greater than 0.6 can be considered as weak, moderate and strong respectively [30, 31].

### Floor and ceiling effects

Floor and ceiling effects for FASH-P were counted as the percentage of participants who scored the extremes. They were considered to be present if more than 15% of individuals scored the lowest (floor) and the highest (ceiling) [32].

### Dimensionality

Since the FASH was introduced as a unidimensional measure, it is important that FASH-P retains these features. Furthermore, the correlation between each item and the total FASH-P score was computed to further assess this psychometric property. The correlation coefficient greater than 0.6 was considered strong [33].

One of the most common ways to assess dimensionality is exploratory factor analysis (EFA). It is the study of strong or weak correlations between a number of sets of items. An EFA with principal component extraction and varimax orthogonal rotation was carried out to determine the number of underlying factors and the number of items loaded on them. Factor extraction was done based on an eigenvalue greater than 1. Substantial importance for a given factor was suggested by items with rotated factor loadings greater than 0.40. In order to assess the appropriateness of EFA for the present data set, the Kaiser-Meyer-Olkin (KMO) value for sampling adequacy and Bartlett’s value for sphericity were considered. The KMO measures how well each variable in a set predicts other variables with no error. This score scores range from 0 to 1, with scores above 0.50 indicating adequate sample size. Also, the Bartlett’s value is used to determine how likely it is that at least some variables in the data set are correlated with each other. A Bartlett’s Significance less than 0.05 ensures that the correlation matrix is not identical and that some relations exist between variables.

Additionally, another parameter to consider in making sure that multicollinearity does not exist in the data for a unidimensional outcome measure is the determinant of the correlation matrix. It should be greater than 0.00001.

### Instruments

Functional Assessment Scale for Acute Hamstring Injuries (FASH).

The FASH is a disease-specific and patient-centered outcome measure developed and validated by Malliaropoulos in 2014 [21]. This questionnaire consists of 10 items to measure the severity of symptoms (pain and disability) in patients with acute hamstring injury. Patient responses are presented on a 0–10 numerical rating except for items 2, 9 and 10 which have a categorical rating system with a specific distribution of 10 points. For each item the number 10 represents the normal level of physical ability and zero represents complete disability. The highest possible score is 100 and the lowest score is theoretically zero. By measuring the impact of HMI on physical function and sport ability, FASH can be used to develop specific rehabilitation programs or to evaluate the treatment efficacy.

### Short‑Form health survey (SF‑36)

The SF-36 questionnaire is a generic tool designed to assess the overall health. This questionnaire includes 36 questions distributed in 8 subscales named general health, physical functioning, role limitation due to physical problem, role limitation due to emotional problem, social functioning, bodily pain, vitality, and mental health. These 8 subscales can be placed in two summary subscales namely physical health and mental health. The final score of this questionnaire is reported as a percentage from 0 to 100. The closer the participant’s score to 100, the better the health status. One question of this questionnaire, separately from the 8 subscales, examines the change in the individual’s health status in the past year. The validity and reliability of this questionnaire has been confirmed in Persian [28].

### Lower extremity functional scale (LEFS)

The LEFS is a patient-centered, region-specific measure of functional status in a wide range of musculoskeletal problems of the lower extremity. This scale has 25 items with a 1–3 Likert scale and provides a functional score as a percentage of normal condition from 0 to 100%. The validity and reliability of the Persian version of this questionnaire was checked and confirmed by Negahban et al. [29].

## Results

The Persian version of the FASH questionnaire was translated exactly according to the original version of the FASH questionnaire without any major problems. The pre-final version was tested by 20 people and there was no problem in understanding the questionnaire, so the pre-final version became the definitive final version.

The total participants of the study sample for the validation phase of FASH-P included 160 people. Of these, they were 40 people (25%) with an acute HMI, 40 people (25%) with another lower limb pathology, 40 people (25%) at risk, and 40 people (25%) controls. The average age in the HMI group was 24.44 (3.25) years, in at risk group it was 23.03 (4.98) years, in the group with other pathologies it was 26.54 (7.34) years, and in the control group it was 23.48 (4.20). No significant difference was observed in the mean age and gender between the groups (all p values were > 0.05). All the participants were professional or recreational athletes and the most represented sports were football, futsal and running. Other sports, such as bodybuilding, combat sports, water sports, volleyball, and handball were also present. The demographic information of the participants is presented in Table 2.

**Table 2:** Demographic characteristic of participants in various evaluated groups

	Hamstring muscle injury	Other injury	At risk	control
Age (year)	24.44(3.25)	26.54(7.34)	23.03(4.98)	23.48(4.20)
Height (cm)	168.12(7.45)	171.31(8.44)	170.36(8.07)	16.7.59(8.8)
Weight (kg)	75.60(9.11)	76.27(8.10)	73.80(8.24)	74.96(11.15)
Hours of training (week)	10.04 (4.91)	7.81(3.93)	12.6(9.27)	7.92(3.45)
Type of sport^*^				
Football and futsal	23(57.5)	17(42.5)	19(47.5)	12(30)
Running	14(35)	8(20)	15(37.5)	11(27.5)
Bodybuilding	2(5)	3(7.5)	0(0)	4(10)
Combat sports	1(2.5)	6(15)	6(15)	8(20)
Other	0(0)	6(15)	0(0)	5(12.5)

* the numbers are frequency (percentile)

The power of differentiation of total score of FASH-P questionnaire out of 100 points was 29.1 ± 14.4 for the pathological group, 91.2 ± 9.8 for the group at risk, 76.9 ± 19.1 for the group with other injuries, and 94.8 ± 6.6 for the control group. Scores were significantly different among the four groups. The mean scores are presented in Table 3.

**Table 3:** Mean ± Standard deviation (number of participants) of the total score of FASH questionnaire obtained in different versions

	Hamstring muscle injury	Other injury	At risk	control
Persian version	29.1 ± 14.4(40)	76.9 ± 19.1(40)	91.2 ± 9.8 (40)	94.8 ± 6.6 (40)
Spanish version	20.6 ± 20.0 (45)	61.6 ± 15.4 (40)	94.9 ± 4.8 (40)	95.3 ± 3.4 (40)
French version	25.6 ± 7.5 (17)	80.8 ± 8.9 (19)	97.8 ± 2.2 (40)	96.6 ± 2.9 (40)
German version	42.7 ± 29.9 (16)	–	90.1 ± 4.7 (19)	97.5 ± 6.3 (77)
English version	25.1 ± 17.6 (40)	74.7 ± 11.9 (30)	97.7 ± 1.6 (40)	98.4 ± 1.8 (30)

Test-retest reliability over a 48 to 60-hour period showed excellent results for all groups for individuals who answered the FASH-P questionnaire twice. In terms of internal consistency, a high level of internal consistency was observed by Cronbach’s alpha of the FASH-P questionnaire (Table 4).

**Table 4:** Test–retest reliability using ICCs for the FASH-P questionnaire

	Hamstring muscle injury	Other injury	At risk	Control	Total	
					ICC	Cronbach’s alpha
**Item 1**	0.98 (0.96–0.99)	0.96 (0.92–0.98)	0.79 (0.54–0.90)	0.96 (0.90–0.98)	0.99 (0.99–0.99)	0.96 (0.95–0.97)
**Item 2**	1.00 (1.00–1.00)	0.96 (0.90–0.98)	0.97 (0.95–0.99)	0.97 (0.93–0.98)	0.97 (0.95–0.98)	0.96 (0.95–0.97)
**Item 3**	0.98 (0.95–0.99)	0.89 (0.74–0.95)	0.79 (0.54–0.90)	0.98 (0.96–0.99)	0.97 (0.95–0.98)	0.96 (0.95–0.97)
**Item 4**	0.99 (0.97–0.99)	0.96 (0.90–0.98)	0.83 (0.59–0.92)	0.86 (0.79–0.99)	0.85 (0.76–0.90)	0.96 (0.95–0.97)
**Item 5**	1.00 (1.00–1.00)	0.99 (0.98–0.99)	0.79 (0.54–0.90)	0.83 (0.74–0.95)	0.99 (0.99–0.99)	0.95 (0.94–0.97)
**Item 6**	0.98 (0.96–0.99)	0.98 (0.97–0.99)	0.98 (0.95–0.99)	0.90 (0.79–0.95)	0.99 (0.98–0.99)	0.96 (0.94–0.97)
**Item 7**	0.99 (0.96–0.99)	0.99 (0.98–0.99)	0.99 (0.98–0.99)	0.93 (0.86–0.97)	0.99 (0.98–0.99)	0.96 (0.94–0.97)
**Item 8**	0.97 (0.95–0.99)	0.97 (0.93–0.98)	1.00 (1.00–1.00)	0.92 (0.83–0.96)	0.99 (0.98–0.99)	0.96 (0.94–0.97)
**Item 9**	1.00 (1.00–1.00)	0.95 (0.89–0.98)	0.96 (0.92–0.98)	0.96 (0.91–0.98)	0.98 (0.97–0.98)	0.96 (0.95–0.97)
**Item 10**	1.00 (1.00–1.00)	0.82 (0.59–0.92)	0.79 (0.54–0.90)	0.95 (0.92–0.98)	0.96 (0.95–0.97)	0.96 (0.94–0.97)
**Total score**	0.99 (0.99–0.99)	0.98 (0.96–0.99)	0.89 (0.76–0.95)	0.92 (0.82–0.96)	0.99 (0.99–0.99)	0.96 (0.95–0.97)

In HMI group, Cronbach’s alpha (after excluding each item) show a high level of internal consistency. Agreement between repeated measurements (SEM) for total score in HMI group was 0.14. The 95% confidence interval of the SEM, as labeled MDC, was 0.38 for this group (Table 5). No participant acquired the minimum or maximum total score in HMI group. In this group for items 2,4,5,9, and 10 more than 15% of the participants obtained minimum score.

**Table 5:** Absolute reliability, internal consistency, and floor and ceiling effect of the FASH-P questionnaire for hamstring muscle injury group

	Cronbach’s alpha*	SEM	MDC	Floor effect	Ceiling effect
**Item 1**	0.954	0.23	0.63	No	No
**Item 2**	0.957	0	0	60%	No
**Item 3**	0.955	0.20	0.55	No	No
**Item 4**	0.961	0.09	0.25	20%	No
**Item 5**	0.965	0	0	60%	No
**Item 6**	0.957	0.18	0.49	No	No
**Item 7**	0.958	0.13	0.36	No	No
**Item 8**	0.958	0.20	0.55	No	No
**Item 9**	0.959	0	0	64%	No
**Item 10**	0.959	0	0	52%	No
**Total score**	0.962	0.14	0.38	No	No

* Cronbach’s alpha after excluding each item

The power of the current study was calculated to be greater than 90% based on the acceptable and expected values for ICC and Cronbach’s alpha values in four different groups and an alpha level of 0.05.

The correlation between the total score of the FASH-P questionnaire and each of the questionnaire’s items was also evaluated. The results showed that all items had a positive and significant correlation with the FASH-P total score (Table 6). In terms of construct validity, FASH-P total score was significantly correlated with SF-36 and LEFS questionnaires. The correlation results of FASH-P total score with LEFS questionnaire and each sub-scale of SF-36 questionnaire are given in Table 6.

**Table 6:** Correlations total FASH-P score with the other measures

	*R*	*p*-value
**FASH**		
Item 1	0.76	< 0.001
Item 2	0.84	< 0.001
Item 3	0.85	< 0.001
Item 4	0.78	< 0.001
Item 5	0.84	< 0.001
Item 6	0.80	< 0.001
Item 7	0.84	< 0.001
Item 8	0.85	< 0.001
Item 9	0.84	< 0.001
Item 10	0.78	< 0.001
**LEFS**	0.66	< 0.001
**SF-36**		
Physical functioning	0.68	< 0.001
Role Physical	0.78	< 0.001
Bodily pain	0.60	< 0.001
General health	0.39	< 0.001
Vitality	0.20	0.041
Social functioning	0.32	0.001
Role emotional	0.71	< 0.001
Mental health	0.16	0.019
Total Physical health	0.69	< 0.001
Total Mental health	0.53	< 0.001
Total score	0.72	< 0.001

FASH: Functional Assessment Scale for Hamstring Injury; LEFS: Lower Extremity Functional Scale; SF-36: Short form-36

Based on exploratory factor analysis, all items loaded on one factor. The Kaiser–Meyer–Olkin was 0.914 which confirms that the sample size is sufficient to carry out factor analysis. The factor model was suitable, as demonstrated by Bartlett’s test of sphericity (approximate Chi square = 1323.693, df = 45, P‹0.001). Using the “eigenvalue > 1” standard, one component explains 80. 05% of the variance. The results of principle component analysis are presented in Table 7.

**Table 7:** Principle component analysis with varimax orthogonal rotation of the FASH-P questionnaire

Item	Factor 1
Item 1	0.930
Item 2	0.892
Item 3	0.938
Item 4	0.761
Item 5	0.950
Item 6	0.922
Item 7	0.910
Item 8	0.935
Item 9	0.771
Item 10	0.915

## Discussion

Having the high rate of recurrence and being responsible for the long period of time spent off the field [1, 6, 9, 11–16] makes HMI a priority of assessment and treatment; therefore, it’s necessary to have a tool available to evaluate the severity of the lesion, its evolution until return to sport and the treatment efficacy. The FASH-P questionnaire was proven to be, reliable, valid and internally consistent to evaluate patients with acute HMI. After carefully following the translation and cross-cultural adaptation process, the final FASH-P was approved by the expert committee, and after first administration, the participants had no difficulty in filling out the questionnaire. Due to the diverse origins of the samples in this study, biases caused by demographic and cultural factors were minimized. In the study of the Greek version of the FASH questionnaire [21], the population was mainly track and field athletes and the study of the German version [22], focused only on football players.

It was observed that the average age of participants in the HMI group was quite young (less than 25 years old), which is in accordance with the available literature [34] and similar to other FASH studies [21–24].

Regarding the FASH-P, psychometric analyses showed that HMI group had lower scores than the other study groups; concluding that this version can differentiate between the pathological group from the group at risk, the group with other injuries and healthy people without a particular risk. This result was similar to previous FASH studies [21–24] showing a coherence between versions even if the German FASH study [22] showed a higher total score, probably due to less diversity of included participants.

The ICC of the total score of FASH-P questionnaire was 0.99 showing excellent test-retest reliability and therefore excellent stability over time. Our results were similar to the French [23], Spanish [24], Greek [21] and German [22] versions of FASH studies that reported high reliability of the questionnaire (ICC above 0.9). evaluating the groups separately, all the results showed high reliability with ICC higher than 0.89.

The standard error of measurement (SEM) and the minimum detectable change (MDC) were calculated to assess the measurement error in the HMI group. The SEM is an estimate of the variation around a true score on repeated measures of a person. The MDC represents the minimal change that a person has to exhibit on a measurement to ensure that the observed changes are true. The SEM and MDC for the FASH-P was 0.14 and 0.38 respectively in the HMI group. The values of SEM (MDC) for the original, Spanish and German versions of FASH were 1.01 (3.05), 2.6(7.2) and 0.78 (2.16) respectively in the HMI group. The SEM for Persian version of the FASH was lesser compared with the other versions. The smaller the SEM, the more precise the measurement capacity of the scale. Regarding the MDC score, there is a 95% confident rate that the functional status of patients with HMI would improve or deteriorate when the change on the FASH-P exceeds 0.38 scale points.

A strong consistency was observed between items of the FASH-P (Cronbach’s alpha higher than 0.96), representing that the items are very sensitive to the evaluated concepts. Other versions of FASH studies also found strong internal consistency (Cronbach’s alpha between 0.917 and 0.980) [21–24]. It should be noted that a very high Cronbach’s alpha value may be an indication of an inefficient level of redundancy in the items. That is to say, the questions may be too similar, leading to the same answers. Internal consistency tells us only about how the items are related to each other, not about whether they are match with each other.

To evaluate the correlation between the items and the total score of the questionnaire, Spearman’s correlation was included. All correlations were statistically significant (a coefficient higher than 0.76) which indicates a strong correlation and, as a result, a very good internal consistency. The French version of FASH study [23] also found a correlation higher than 0.6 between all items and total score of the questionnaire.

In terms of the criterion validity, a strong correlation was found between the FASH-P and the LEFS and SF-36 questionnaires. The degree of correlation with LEFS was found to be 0.66, indicating a strong correlation. A high correlation was also found between the FASH-P and the total score of SF-36 (0.72), Although the degree of correlation was not the same for all subscales of SF-36 and the correlation level was weak for some subscales (General health, Mental health and Vitality). This result was consistent with the results of the French [23] and German [22] versions of the questionnaire. In the study of the Greek version [21], the criterion validity was determined using the VISA-H questionnaire, which showed good criterion validity.

Based on factor analysis, just one factor explained 80.05% of the variance within the FASH-P and all items loaded on one factor. It confirms that there was a unique dimension. This was in line with finding of original and Spanish version of FASH which achieved a unique factor solution that explained 95.8% and 80% of the variance respectively [21, 24]. Such results confirm the construct validity of the FASH-P and the use of single-sum score for this outcome measure, which makes its clinical use reasonable. This property was not considered in the French and German version of the FASH.

### Clinical implications

The FASH-P has been shown to be a discriminant, reliable, and valid instrument for evaluating Persian-speaking patients with acute hamstring injuries. This tool may help clinicians and researchers in evaluating the impact of hamstring muscle injuries on the functional capacity of Persian-speaking patients and documenting the effectiveness of treatments. This could facilitate improved planning for rehabilitation and return to sport.

### Limitations and future perspectives

Due to the cross-sectional design of the study, the responsiveness of the FASH-P was not assessed that should be considered in future projects enabling better planning of rehabilitation and making return to sport decisions.

## Conclusions

Due to the excellent psychometric performance, the FASH-P can be used as a reliable and valid tool for evaluating the severity of symptoms and sports ability in Persian-speaking patients with hamstring muscle injury.

## Data Availability

The datasets used and/or analyzed during the current study are available from the corresponding author on reasonable request.

## Abbreviations

FASH: Functional Assessment Scale for Acute Hamstring Injuries
FASH-P: Persian version of FASH questionnaire
SF-36: Short Form Health Survey
LEFS: Lower Extremity Functional Scale
HMI: Hamstring muscle injury
WHO: World Health Organization
IRB: Institutional Review Board
ICC: Intra-class correlation coefficient
SEM: Standard error of measurement
MDC: Minimal detectable change
LLFI: Lower limb functional index
EFA: Exploratory factor analysis
KMO: Kaiser-Meyer-Olkin

## References

[1] Brooks J, Fuller C, Kemp S. Incidence, risk, and prevention of hamstring muscle injuries in professional rugby union. Br J Sports Med. 2007;41:543–4. 10.1177/0363546505286022 16493170

[2] Ekstrand J, Hägglund M, Waldén M. Epidemiology of muscle injuries in professional football (soccer). Am J Sports Med. 2011;39(6):1226–32. 21335353 10.1177/0363546510395879

[3] Feddermann-Demont N, Junge A, Edouard P, Branco P, Alonso J-M. Injuries in 13 international Athletics championships between 2007–2012. Br J Sports Med. 2014;48(7):513–22. 24620039 10.1136/bjsports-2013-093087

[4] Malliaropoulos N, Papacostas E, Kiritsi O, Rad P-M, Papalada A, Gougoulias N, et al. Posterior thigh muscle injuries in elite track and field athletes. Am J Sports Med. 2010;38(9):1813–9. 20522825 10.1177/0363546510366423

[5] Meeuwisse WH, Sellmer R, Hagel BE. Rates and risks of injury during intercollegiate basketball. Am J Sports Med. 2003;31(3):379–85. 12750130 10.1177/03635465030310030901

[6] Orchard J, Best TM, Verrall GM. Return to play following muscle strains. Clin J Sport Med. 2005;15(6):436–41. 16278548 10.1097/01.jsm.0000188206.54984.65

[7] Orchard J, Seward H. Epidemiology of injuries in the Australian Football League, seasons 1997–2000. Br J Sports Med. 2002;36(1):39–44. 11867491 10.1136/bjsm.36.1.39 PMC1724448

[8] Kakavas G, Malliaropoulos N, Kaliakmanis A, Georgios B, Maffulli NJJBRHA. A ninety-minute football match increases hamstring flexibility in professional players. J Biol Regul Homeost Agents. 2020;34(5):87–92. 33739011

[9] Ekstrand J, Waldén M, Hägglund M. Hamstring injuries have increased by 4% annually in men’s professional football, since 2001: a 13-year longitudinal analysis of the UEFA Elite Club injury study. Br J Sports Med. 2016;50(12):731–7. 26746908 10.1136/bjsports-2015-095359

[10] Lempainen L, Banke IJ, Johansson K, Brucker PU, Sarimo J, Orava S, et al. Clinical principles in the management of hamstring injuries. Knee Surg Sports Traumatol Arthrosc. 2015;23(8):2449–56. 24556933 10.1007/s00167-014-2912-x

[11] Croisier J-L. Factors associated with recurrent hamstring injuries. Sports Med. 2004;34(10):681–95. 15335244 10.2165/00007256-200434100-00005

[12] Ekstrand J, Hägglund M, Waldén M. Injury incidence and injury patterns in professional football: the UEFA injury study. Br J Sports Med. 2011;45(7):553–8. 19553225 10.1136/bjsm.2009.060582

[13] Hägglund M, Waldén M, Ekstrand J. Previous injury as a risk factor for injury in elite football: a prospective study over two consecutive seasons. Br J Sports Med. 2006;40(9):767–72. 16855067 10.1136/bjsm.2006.026609 PMC2564391

[14] Hallén A, Ekstrand J. Return to play following muscle injuries in professional footballers. J Sports Sci. 2014;32(13):1229–36. 24784885 10.1080/02640414.2014.905695

[15] Petersen J, Hölmich P. Evidence based prevention of hamstring injuries in sport. Br J Sports Med. 2005;39(6):319–23. 15911599 10.1136/bjsm.2005.018549 PMC1725237

[16] Hickey J, Shield AJ, Williams MD, Opar DA. The financial cost of hamstring strain injuries in the Australian Football League. Br J Sports Med. 2014;48(8):729–30. 24124035 10.1136/bjsports-2013-092884

[17] Malliaropoulos N, Isinkaye T, Tsitas K, Maffulli NJTA. Reinjury after acute posterior thigh muscle injuries in elite track and field athletes. Am J Sports Med. 2011;39(2):304–10. 21051422 10.1177/0363546510382857

[18] Engebretsen AH, Myklebust G, Holme I, Engebretsen L, Bahr R. Intrinsic risk factors for groin injuries among male soccer players: a prospective cohort study. Am J Sports Med. 2010;38(10):2051–7. 20699426 10.1177/0363546510375544

[19] McGregor C, Ghosh S, Young DA, Maffulli NJD. Rehabilitation. Traumatic and overuse injuries of the ischial origin of the hamstrings. Disabil Rehabil. 2008;30(20–22):1597–601. 18608384 10.1080/09638280701786138

[20] van der Horst N, van de Hoef S, Reurink G, Huisstede B, Backx FJSM. Return to play after hamstring injuries: a qualitative systematic review of definitions and criteria. Sports Med. 2016;46:899–912. 26767837 10.1007/s40279-015-0468-7 PMC4887544

[21] Malliaropoulos N, Korakakis V, Christodoulou D, Padhiar N, Pyne D, Giakas G, et al. Development and validation of a questionnaire (FASH—Functional Assessment Scale for Acute Hamstring injuries): to measure the severity and impact of symptoms on function and sports ability in patients with acute hamstring injuries. Br J Sports Med. 2014;48(22):1607–12. 25287515 10.1136/bjsports-2014-094021

[22] Lohrer H, Nauck T, Korakakis V, Malliaropoulos N. Validation of the FASH (Functional Assessment Scale for Acute Hamstring injuries) questionnaire for german-speaking football players. J Orthop Surg Res. 2016;11(1):130. 27776547 10.1186/s13018-016-0464-0 PMC5078932

[23] Locquet M, Willems T, Specque C, Beaudart C, Bruyère O, Van Beveren J et al. Cross-cultural adaptation, translation, and validation of the functional assessment scale for acute hamstring injuries (FASH) questionnaire for french-speaking patients. Disabil Rehabil. 2018:1–7. 10.1080/09638288.2018.1544669 30669878

[24] Hernández-Sanchez S, Korakakis V, Malliaropoulos N, Moreno-Perez V. Validation study of the Functional Assessment Scale for Acute Hamstring injuries in Spanish professional soccer players. Clin Rehabil. 2018:0269215518815540. 10.1177/0269215518815540 30526019

[25] Organization WH. Process of translation and adaptation of instruments. http://www.whoint/substance_abuse/research_tools/translation/en/. 2009.

[26] Tavakol M, Dennick R. Making sense of Cronbach’s alpha. Int J Med Educ. 2011;2:53. 28029643 10.5116/ijme.4dfb.8dfd PMC4205511

[27] Munro BH. Statistical methods for health care research. lippincott williams & wilkins; 2005.

[28] Montazeri A, Goshtasebi A, Vahdaninia M, Gandek B. The short Form Health Survey (SF-36): translation and validation study of the Iranian version. Qual Life Res. 2005;14:875–82. 16022079 10.1007/s11136-004-1014-5

[29] Negahban H, Hessam M, Tabatabaei S, Salehi R, Sohani SM, Mehravar M. Reliability and validity of the Persian lower extremity functional scale (LEFS) in a heterogeneous sample of outpatients with lower limb musculoskeletal disorders. Disabil Rehabil. 2014;36(1):10–5. 23530691 10.3109/09638288.2013.775361

[30] Fayers PM, Machin D. Quality of life: the assessment, analysis and interpretation of patient-reported outcomes. Wiley; 2013.

[31] Hinkel DE, Wiersma W, Jurs SG. Applied statistics for the behavioral sciences. Houghton Mifflin Co; 1988.

[32] Ware JE Jr, Gandek BJJ. Methods for testing data quality, scaling assumptions, and reliability: the IQOLA Project approach. 1998;51(11):945 – 52. 10.1016/s0895-4356(98)00085-7 9817111

[33] Taylor R. Interpretation of the correlation coefficient: a basic review. J Diagn Med Sonography. 1990;6(1):35–9.

[34] Woods C, Hawkins R, Maltby S, Hulse M, Thomas A, Hodson A. The Football Association Medical Research Programme: an audit of injuries in professional football—analysis of hamstring injuries. Br J Sports Med. 2004;38(1):36–41. 14751943 10.1136/bjsm.2002.002352 PMC1724733

